# Thromboembolism in renal transplant artery due to atrial fibrillation

**DOI:** 10.5414/CNCS108029

**Published:** 2013-10-28

**Authors:** Janina Müller-Deile, Anke Schwarz, Jan Menne

**Affiliations:** Division of Nephrology and Hypertension, Department of Medicine, Hannover Medical School, Hannover, Germany

**Keywords:** atrial fibrillation, kidney transplantation, thromboembolization

## Abstract

Background: Central and peripheral arterial thromboembolisms are well known complications of atrial fibrillation. We report the first case of thromboembolism in a renal transplant due to atrial fibrillation after changing anticoagulation treatment. Case presentation: A 79-year-old woman who had undergone kidney transplantation in 2001 presented herself with abdominal pain and oliguria. Serum creatinine had been stable (130 – 150 µmol/l) since transplantation, and, because of atrial fibrillation, the patient received oral anticoagulation treatment for many years until it was switched to aspirin 100 mg due to a cholecystectomy in 2012. Three weeks thereafter is when the patient was admitted to our unit. In a computed tomography scan of the abdomen, multiple renal infarcts were detected. The thromboembolism resulted in a complete loss of transplant function. As the patient remained anuric, the transplant was declared to be lost, immunosuppression was reduced, and renal replacement therapy was commenced over a central catheter. However, the patient died the next day. Conclusion: Our case highlights the fact that changing anticoagulation treatment should be performed carefully and can be detrimental. The location of thromboembolism in renal transplant artery makes this case unique and has not ever been described thus far. As renal transplantation and risk factors for thromboembolism i.e., atrial fibrillation are increasing, embolization in renal transplant artery is a potential reason for abdominal pain and anuria; physicians should take this into consideration when treating transplanted patients.

## Background 

Stroke and less common peripheral arterial thromboembolism are well known complications of atrial fibrillation [1, 2]. The reason for unequal incidence of thromboembolism in different organs is unknown. Renal infarcts of native kidney due to embolism are often difficult to diagnose and are probably underestimated [3, 4]. Reviewing the literature, peripheral arterial thromboembolism in a kidney transplant artery has never been described to date. 

## Case presentation 

We report the case of a 79-year-old woman who received a kidney transplant in 2001 and maintained good function thereafter. The primary kidney disease was not known. In October 2012, she presented to our emergency unit with a history of abdominal pain, fever (38 °C), and shivering. Two days prior, urinary excretion had decreased to 600 ml/d. On the day of presentation, the patient was anuric. She described her abdominal pain as being similar to that of cholecystitis from which she had suffered before. A cholecystectomy had been performed 3 weeks prior in another hospital. 

Her past medical history included atrial fibrillation, coronary heart disease (with coronary artery bypass graft in 2006), stroke, epilepsy (since childhood), and hypertension. Her immunosuppressive medication consisted of mycophenolate and cyclosporine. She was taking candesartan, torasemid, metoprolol, and amlodipine for hypertension, and as an anti-epileptic prophylaxis, lamotrigine. Oral anticoagulation was discontinued and replaced by aspirin 100 mg prior to the cholecystectomy. 

Upon admission, her vital signs were as follows: blood pressure of 123/70 mmHg and heart rate of 61 beats per minute. There was tenderness and pain on palpitation in the region of the kidney transplant in the left inguinal area. The other examination was unremarkable. 

**Figure 1.**
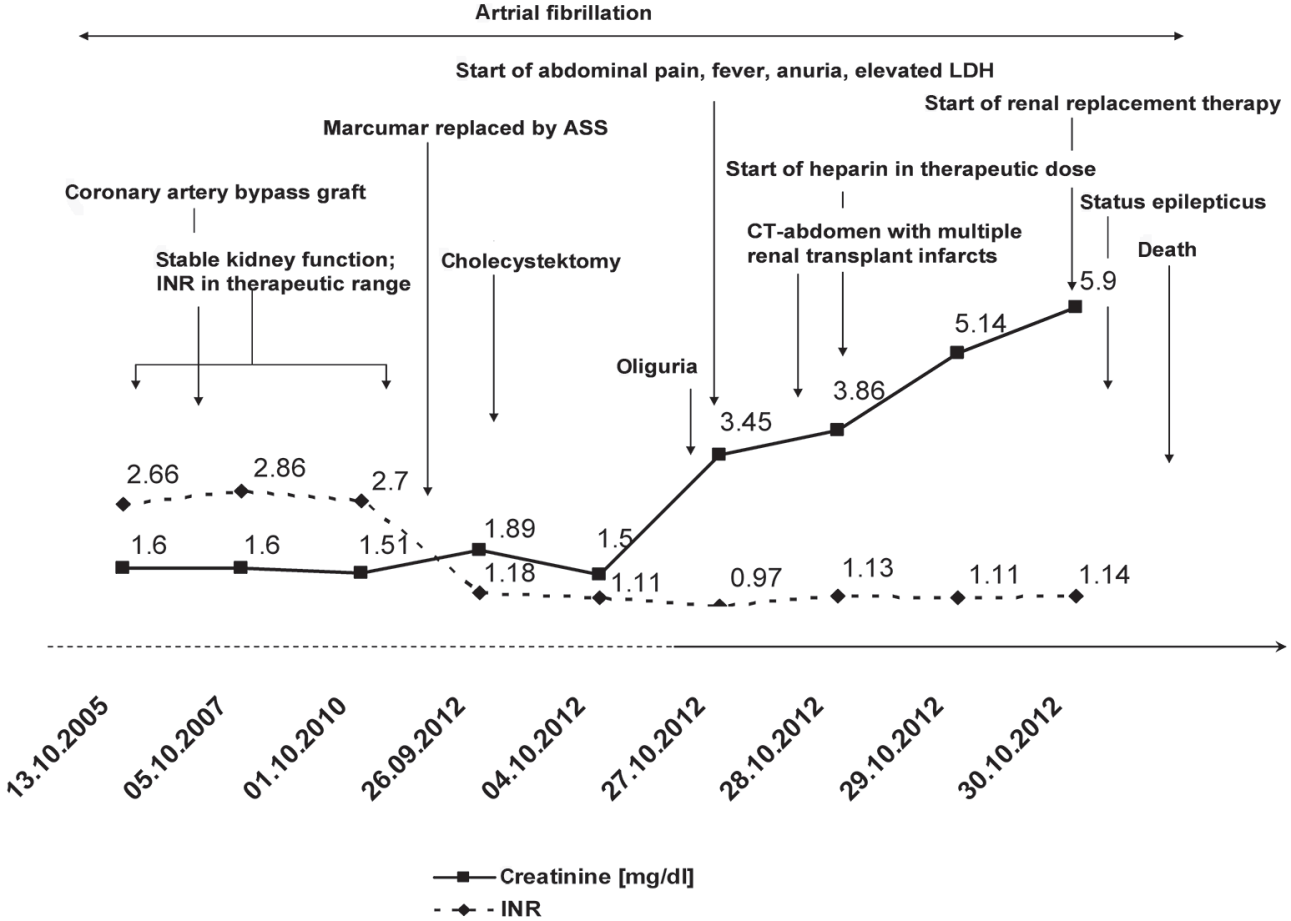
Patient’s clinical history. Illustration of patient’s creatinine and INR over time and hallmarks of clinical history.

Laboratory results revealed a white blood count of 17.5 × 10^9^/l (being in the normal range before), erythrocytes of 3.49 × 10^9^/l and platelets of 148 × 10^9^/l. C-reactive protein was 33 mg/l, potassium was in the normal range, but creatinine was elevated to 304 µmol/l (3.45 mg/dl). Previous mean creatinine levels had been ~ 140 µmol/l (1.58 mg/dl) since renal transplantation in 2001. Lactate dehydrogenase (LDH) was strongly elevated to 1,382 U/dl (nl < 250 U/dl). International normalized ratio (INR) was in normal range as oral anticoagulation with Marcumar was stopped, pending cholecystectomy. Before, the patient’s INR had been in the therapeutic range for years (Figure 1). 

**Figure 2.**
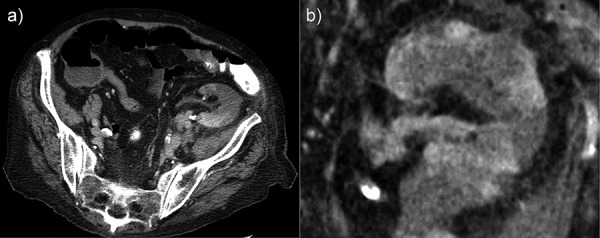
Computed tomography of patient’s abdomen. Axial (a) and coronary (b) computed tomography with contrast media showing several large infarct areas in the transplanted kidney (left iliac fossa). The renal artery is perfused. Slide size of computed tomography is 1.25 mm.

Electrocardiogram showed atrial fibrillation with 70 beats per minute. X-ray of the chest and abdomen could neither detect infiltrates nor free gas or free fluid. An antibiosis with ertapenem was started after blood cultures had been taken. A computed tomography (CT) scan of the abdomen, with contrast, revealed multiple infarct areas affecting at least 50 – 60% of the transplanted parenchyma (Figure 2). We began anticoagulation therapy with heparin in therapeutic doses. Over the following days, creatinine levels increased to 522 µmol/l (5.9 mg/dl). As the patient remained anuric, the transplant was declared to be lost, immunosuppression was reduced, and renal replacement therapy was commenced over a central catheter. 

The following day, the patient developed a status epilepticus that did not respond to high doses of anti-epileptic drugs. A cranial CT ruled out intracranial bleeding or a mass. No fresh infarct was noted. Cranial MRI was not possible because of the patient’s agitation. The patient died within 24 hours in our intensive care unit after a decision was made in consensus with her relatives not to intensify the treatment. An autopsy was refused by the family. 

## Discussion 

The incidence of peripheral arterial thromboembolic events in patients with atrial fibrillation is much lower than the incidence of strokes [1]. 

The most common sites for peripheral arterial thromboembolism are the extremities (61%), followed by the mesenteric arteries (29%), the pelvic arteries (9%), the aorta (7%), and the renal arteries (2%) [2]. Peripheral arterial thromboembolic events causing renal transplant damage seem to be exceptional as we could not find any similar published cases. One explanation for the rarity might be the unlikelihood for thrombi reaching renal transplant artery due to anatomical reasons. 

Renal infarction of native kidney occurs in patients with high morbidity and mortality and is associated with diffuse atherosclerosis and atrial fibrillation [3]. It is often difficult to diagnose because the symptoms are similar to those of nephrolithiasis, pyelonephritis, ruptured abdominal aortic aneurysm, testicular/ovarian torsion, incarcerated hernia, intestinal perforation, mesenteric ischemia, diverticulitis, spinal disease, or myocardial infarction. Thus, as it shown in post-mortem studies, the real incidence of renal artery embolism is probably underestimated [4]. In our case, abdominal CT together with the patient’s medical history and laboratory results led to the diagnosis. 

As renal artery was perfused in our patient, we had no evidence for local stenosis. “Cortical rim sign” (1- to 3-mm rim of subcapsular enhancement paralleling the renal margin) is a common feature found with renal artery obstruction by CT [5]. “Arterial cut-off-sign” (abrupt termination of contrast material in the renal artery) [6] and “flip-flop enhancement” (delayed enhancement of the initial hypo attenuations due to extravasation of contrast material in areas of ischemia) [7] are other possible features in CT. 

Frequently, patients with renal infarction of native kidney are initially hypertensive, which is thought to be renin-mediated due to decreased renal blood supply [3]. Our patient’s blood pressure was normal under multiple antihypertensive agents. Urine analysis after renal infarction of native kidney is associated with hematuria [8]. Proteinuria was seen in 45% of the patients in one retrospective case review [9]. Elevations of LDH are almost universal in renal infarction, and LDH can also be detected in urine [10, 11]. The rise in LDH has been considered as a good screening marker in patients suffering from embolism [12]. Our patient was anuric. Therefore, no urine assessment for LDH, hemoglobin, or protein was possible. Another typical finding in renal artery embolism is the elevation in white blood cell count [5], a fact that was also seen in our patient. 

Typical signs, symptoms, and laboratory findings associated with atheroembolic renal disease (AERD) due to cholesterol embolism such as livedo reticularis, gangrene of the extremities, and neurological symptoms like amaurosis fugax or stroke-like episodes or eosinophilia were not observed. Furthermore, the patient had not received an intervention that would have triggered the occurrence of AERD. Therefore, we have no evidence that kidney failure was caused by cholesterol embolism. 

In prior cases of artery embolism of the native kidney, serum creatinine was not markedly elevated at presentation. In a study of patients with 44 cases of renal infarction with atrial fibrillation by Hazanov et al. [9], 61% had normal renal function on follow-up. In our case, the patient was dependent on renal transplant since native kidneys had been removed years before. Thus, severe thromboembolism of renal transplant artery resulted in complete loss of kidney function and necessity for renal replacement therapy. 

A known treatment option of artery embolism is anticoagulation. Thrombolysis or thrombectomy can be considered, but no significant clinical evidence supports these interventions. For renal transplant embolism resulting in renal transplant infarction, no special treatment option has yet been described. 

## Conclusions 

Our case highlights the fact that changing anticoagulation treatment should be performed carefully as it can be detrimental. In our patient, the location of thromboembolism was the renal transplant artery; this resulted in complete loss of kidney transplant. 

As renal transplantation and risk factors for thromboembolism i.e., atrial fibrillation are increasing, embolization in renal transplant artery is a potential reason for abdominal pain and anuria, physicians should take this into consideration when treating transplanted patients. 

Until now, the only known treatment option is anticoagulation, and thus far no special treatment option has been described. Therefore, research in this field is highly requested. 

## Consent 

Written informed consent for publication of the clinical details and clinical images was obtained from the relatives of the patient. 

## Competing interests 

The authors declare that they have no competing interests.
